# Reelin Can Modulate Migration of Olfactory Ensheathing Cells and Gonadotropin Releasing Hormone Neurons via the Canonical Pathway

**DOI:** 10.3389/fncel.2018.00228

**Published:** 2018-08-03

**Authors:** Leigh Dairaghi, Ellen Flannery, Paolo Giacobini, Aybike Saglam, Hassan Saadi, Stephanie Constantin, Filippo Casoni, Brian W. Howell, Susan Wray

**Affiliations:** ^1^Cellular and Developmental Neurobiology Section, National Institute of Neurological Disorders and Stroke (NINDS), National Institutes of Health (NIH), Bethesda, MD, United States; ^2^Coriell Institute for Medical Research, Camden, NJ, United States; ^3^Laboratory of Development and Plasticity of the Neuroendocrine Brain, Jean Pierre Aubert Research Centre, INSERM U1172, Lille, France; ^4^Division of Neuroscience, San Raffaele Scientific Institute, Università Vita-Salute San Raffaele, Milan, Italy; ^5^Neuroscience and Physiology, Upstate Medical University, Syracuse, NY, United States

**Keywords:** GnRH, LHRH, reelin, migration, olfactory system, olfactory ensheathing cells, Dab1

## Abstract

One key signaling pathway known to influence neuronal migration involves the extracellular matrix protein Reelin. Typically, signaling of Reelin occurs via apolipoprotein E receptor 2 (ApoER2) and very low-density lipoprotein receptor (VLDLR), and the cytoplasmic adapter protein disabled 1 (Dab1). However, non-canonical Reelin signaling has been reported, though no receptors have yet been identified. Cariboni et al. (2005) indicated Dab1-independent Reelin signaling impacts gonadotropin releasing hormone-1 (GnRH) neuronal migration. GnRH cells are essential for reproduction. Prenatal migration of GnRH neurons from the nasal placode to the forebrain, juxtaposed to olfactory axons and olfactory ensheathing cells (OECs), has been well documented, and it is clear that alterations in migration of these cells can cause delayed or absent puberty. This study was initiated to delineate the non-canonical Reelin signaling pathways used by GnRH neurons. Chronic treatment of nasal explants with CR-50, an antibody known to interfere with Reelin homopolymerization and Dab1 phosphorylation, decreased the distance GnRH neurons and OECs migrated. Normal migration of these two cell types was observed when Reelin was co-applied with CR-50. Immunocytochemistry was performed to determine if OECs might transduce Reelin signals via the canonical pathway, and subsequently indirectly altering GnRH neuronal migration. We show that in mouse: (1) both OECs and GnRH cells express ApoER2, VLDLR and Dab1, and (2) GnRH neurons and OECs show a normal distribution in the brain of two mutant *reeler* lines. These results indicate that the canonical Reelin pathway is present in GnRH neurons and OECs, but that Reelin is not essential for development of these two systems *in vivo*.

## Introduction

Neuronal migration relies on complex networks of intracellular signaling pathways, and activation of these pathways is orchestrated by spatially and temporally distributed guidance cue ligands and their receptors. Reelin, a glycoprotein of the extracellular matrix (ECM; D’Arcangelo et al., 1995; Rice and Curran, 2001) regulates neuronal positioning during cerebral development (Tissir and Goffinet, 2003; Katsuyama and Terashima, 2009). Mice with homozygous *reelin* gene mutations, *reeler* (Reln^rl^), develop a disorganized cortex in which late-born neurons fail to migrate past earlier-born neurons (D’Arcangelo et al., 1995; for review, see Rice and Curran, 2001; Tissir and Goffinet, 2003; Katsuyama and Terashima, 2009). Proposed functions of Reelin include stop, detachment, permissive, attractive, branch-inducing and laminar targeting signals (Tissir and Goffinet, 2003; Soriano and Del Río, 2005; Luque, 2007; Cooper, 2008). All of these processes are involved in neurons reaching their appropriate target location.

The canonical pathway first identified for Reelin was binding to very low-density lipoprotein receptor (VLDLR) and apolipoprotein E receptor 2 (ApoER2; Hiesberger et al., 1999). Binding of Reelin to these receptors leads to the phosphorylation, downstream signaling, and subsequent degradation of the adaptor protein Disabled 1 (Dab1; D’Arcangelo et al., 1999; Hiesberger et al., 1999; Howell et al., 1999; Arnaud et al., 2003). Inactivation of Dab1 by gene targeting or spontaneous mutation caused a *reeler*-like disorganized cortex (Sheldon et al., 1997; Arnaud et al., 2003) consistent with the Dab1 docking protein being an obligate component of the Reelin signaling pathway (Sheldon et al., 1997; Arnaud et al., 2003). However, non-canonical Reelin signaling has been reported (for review, see Bock and May, 2016). Of the proposed models, only two do not require Dab-1, with one of these models using alternative receptors as well (Bock and May, 2016). Examples of Dab-1 independent signaling include: (1) a Reelin-dependent effect on the migration of early-generated interneurons in the olfactory bulb, which was defective in lipoprotein receptor-deficient mice but was not phenocopied in mice lacking Dab1 (Hellwig et al., 2012); (2) hypothalamic Gonadotropin Hormone-Releasing Hormone-1 (GnRH)-positive neurons, with a reduction in number and aberrant position of GnRH neurons in the hypothalamus of *reeler*, but not Dab1 or ApoER2/VLDLR- deficient mice (Cariboni et al., 2005); and (3) changes in lymphatic vascular development, which is defective in *reeler* mice but not in mice lacking Dab1 or both ApoER2 and VLDLR (Lutter et al., 2012). In none of these cases were the molecular mechanisms underlying the described Reelin-dependent phenotypes elucidated.

In vertebrates, reproduction is dependent on hypothalamic neurons secreting the neuropeptide GnRH, which regulates anterior pituitary gonadotropes and thus gonadal function. During embryonic development, GnRH neurons differentiate in the nasal placode and migrate to the hypothalamus apposed to olfactory-vomeronasal nerves (Schwanzel-Fukuda and Pfaff, 1989; Wray et al., 1989) and olfactory ensheathing cells (OECs). In humans, several monogenic, as well as digenic, disorders leading to idiopathic hypogonadotropic hypogonadisms (IHH) are caused by disruption of GnRH neuronal ontogeny/migration (Pitteloud et al., 2007). Identifying molecules regulating the development of the GnRH system will facilitate understanding pathogenesis of human IHH disorders as well as developmental processes involved in neuronal movement.

The present study was initiated to delineate the non-canonical Reelin signaling pathway used by GnRH neurons. Unexpectedly, VLDLR, ApoER2 and the adaptor protein Dab1 were found in OECs and GnRH neurons during embryonic mouse development. No defects in GnRH neuronal migration, olfactory axons and/or OECS entering the olfactory bulb, were found in *reeler* mice either prenatally or postnatally. However, chronic immunodepletion of Reelin in explants decreased both GnRH cell and OECs migration distance and this attenuation was rescued by co-application of Reelin. These data indicate that the Reelin pathway can modulate, but is not essential, for the development of GnRH neurons and OECs, and that both of these cell types possess the necessary components for Reelin signaling through the canonical pathway and as such, are not an appropriate model for identifying a non-conventional Reelin signaling pathway.

## Materials and Methods

### Animals

All procedures were approved by National Institute of Neurological Disorders and Stroke (NINDS) ACUC and performed in accordance with National Institutes of Health (NIH) guidelines.

*In vitro*: embryos were obtained from timed pregnant NIH Swiss mice at E11.5. Nasal explants devoid of brain tissue were generated, cultured and maintained as previously described (Fueshko and Wray, 1994; Klenke and Taylor-Burds, 2012). Briefly, nasal pits were dissected under aseptic conditions in Gey’s balanced salt solution (Life Technologies Inc., Grand Island, NY, USA) supplemented with glucose (Sigma Chemical Co., St. Louis, MO, USA). Explants were adhered onto coverslips by a plasma (Cocalico Biologicals, Reamstown, PA, USA)/thrombin (Sigma) clot and maintained in a defined serum-free medium (SFM; Wray et al., 1991) in a humidified atmosphere at 37°C with 5% CO_2_. On culture day 3, SFM was replaced by fresh SFM containing fluorodeoxyuridine (2.3 μM; Sigma) for 3 days to inhibit proliferation of dividing olfactory neurons and non-neuronal explant tissue.

*In vivo*: *reeler* mice were purchased from Jackson Laboratories (B6C3Fe a/a-Reln/J). *Reeler* and wild-type embryos (E13.5-E14.5; plug day, E0.5), as well as brains harvested at 26, 35 and 90 days after birth (≥PN26), were fresh-frozen in dry-ice and stored (−80°C) until processing for immunohistochemistry. For double-immunofluorescence, embryos were fixed overnight at 4% PFA (4% paraformaldehyde in 0.1 M phosphate buffer, pH 7.4) and cryoprotected, then frozen and stored (−80°C) until processing. In addition, perfused adult *reeler*/Orleans brains were obtained from Dr. P. Phelps (UCLA). GnRH-GFP mice (Spergel et al., 1999) were used for *in vivo* colocalization immunofluorescence experiments.

### Immunocytochemistry

Primary antisera were rabbit polyclonal unless otherwise indicated, and were directed against: GnRH (SW, 1:3,000 for chromogen-staining and 1:1,000 for immuno-fluorescence (Wray et al., 1988); mouse monoclonal (1:4,000, gift from Dr. Karande Gangatirkar et al., 2002), VLDLR (1:2,000, goat polyclonal; R&D system), Reelin (1:1,000, mouse IgG monoclonal G10, gift of Dr. A. Goffinet de Bergeyck et al., 1997), ApoER2 (1:500, gift of Dr. J. Herz Beffert et al., 2004), Dab1 (1:1,000 (Howell et al., 1997) and 1:1,000; Rockland Immunochemicals Inc.), βtubulin III (TUJ1, 1:1,000, mouse IgG, Sigma), p75 nerve growth factor receptor (p75NGF, 1:5,000, Chemicon), tyrosine hydroxylase (TH, 1:700, PelFreez), green fluorescent protein (GFP, 1:1,000, chicken polyclonal, Abcam), S100 (1:4,000, Dako), Sox10 (1:150, goat polyclonal, Santa Cruz) and peripherin (1:1,000, Chemicon).

Mouse tissue sections (Forni et al., 2011) or explants (Kramer et al., 2000) were stained for GnRH as previously described. Mouse embryos and ≥PN26 brains were cryosectioned at 16 μm and 35 μm, respectively. Briefly, fresh-frozen sections and explants were fixed with 4% formaldehyde in phosphate buffered saline (PBS, 1 h), washed (6×PBS), incubated in 10% normal horse serum/0.3% Triton X-100 (NHS/Tx-100, 1 h), washed several times in PBS, and placed in primary antibody (overnight 4°C). The next day, tissues were washed (PBS), incubated in biotinylated secondary antibody (1 h; 1:500 in PBS/0.3% Triton X-100; anti-rabbit biotinylated (Vector Laboratories Inc, Burlingame, CA, USA; anti-mouse biotinylated, Chemicon) and processed using a standard avidin-biotin-horseradish peroxidase/3’,3-diaminobenzidine (DAB or enhanced nickel DAB) protocol. For double immunoperoxidase staining the chromogen for the first antigen–antibody complex was DAB (brown precipitate), whereas the chromogen for the second antigen-antibody complex was SG substrate (blue precipitate, Vector). For double-immunofluorescence experiments, fixed embryonic sections (14 μm) or explants were fixed in 4% formaldehyde (10 min sections, 1 h explants), washed in PBS, blocked (1 h, NHS/Tx-100), washed, and incubated in primary antibody (anti-ApoER2, anti-Dab1, or anti-VLDL; two nights at 4°C). Thereafter, they were washed and placed in fluorescent secondary (1 h, 1:1,000, Alexa Fluor-555, Life Technologies), washed, and fixed briefly. For double labeling with primary antibodies produced in the same species (rabbit), sections were washed, incubated (30 min normal rabbit serum (10%)/0.3% Triton X-100), washed and then blocked with anti-rabbit IgG FAB fragment (80 μg/ml in BSA, Jackson) for at least 1 h. After washing in PBS, sections were fixed briefly, washed, and then incubated in the second primary antibody (anti-S100; two nights at 4°C). The next day, sections were washed and incubated in fluorescent secondary (1 h, 1:1,000, Alexa Fluor-488 Invitrogen). After fluorescent labeling, embryo sections with high levels of blood vessel autofluorescence were treated with TrueBlack (Whittington and Wray, 2017; Biotium; 3 min). All slides were coverslipped with Vectashield hardset mounting medium (Vector). DAPI treatment either occurred immediately prior to coverslipping or was included in the mounting medium. Controls: omission of the first antibody produced no detectable signal (data not shown). The efficiency of the anti-rabbit FAB fragment blocker was verified by omission of the second primary antibody with all other conditions remaining the same. Application of the second fluorescent secondary antibody resulted in no double-labeled structures (data not shown) and glial markers did not stain GnRH cells when both were stained using rabbit antibodies (see Figure 4C).

**Figure 1:**
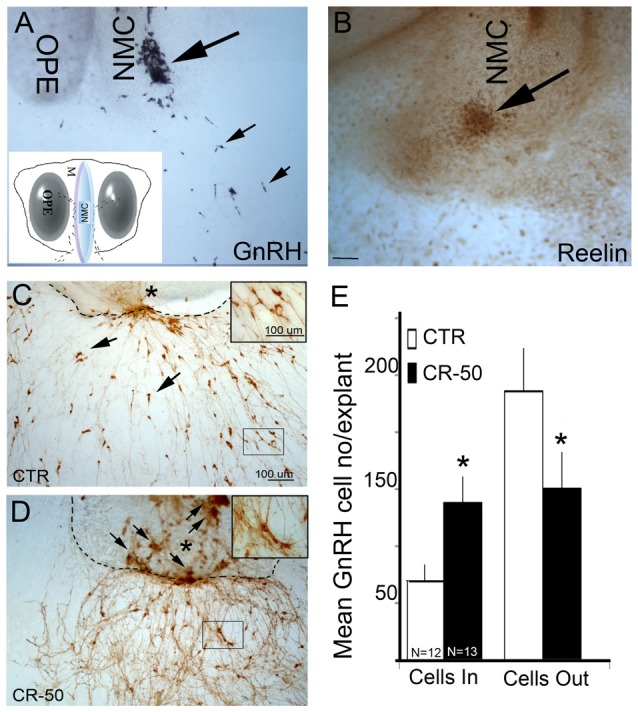
Reelin signaling blockade *in vitro* decreases gonadotropin releasing hormone-1 (GnRH) neuronal migration. **(A)** GnRH cells (blue-black, small arrows) have migrated into the periphery of the explant by 3 *div*, although large numbers remain at the tip of the nasal midline cartilage (NMC, large arrow). Inset: schematic of nasal explant (OPE, olfactory pit epithelium). **(B)** Immunostaining with antibody against Reelin in a 3 *div* explant, shows strong expression of Reelin (brown, arrow) at the tip of NMC. Reelin is expressed by mesenchymal cells in the area where GnRH neurons migrate into the periphery (Compare large arrows in **A–D**). 7 *div* explants immunostained with antibody against GnRH (brown) after chronic treatment (from 3–6 *div*) either with vehicle (CTR) **(C)** or CR-50 antibody **(D)**, an antibody known to block the effect of Reelin. Dash lines indicate border between the main tissue mass and the periphery of the explant, asterisk indicates tip of NMC. Under control (CTR) conditions, GnRH-positive cells are dispersed outside the main tissue mass (**C**, arrows), in the periphery of the explants. After CR-50 treatment, the majority of the GnRH neurons remained within the main tissue mass (**D**, arrows) and fewer cells migrated into the periphery. **(E)** Quantitative analysis of GnRH cell distribution in the inner tissue mass and in the periphery under the two experimental conditions. The total number of GnRH cells did not change between control and treated explants. However, following CR-50 treatment significantly more GnRH neurons remained within the inner tissue mass (Cells In) as compared to control conditions (*P < 0.01; Student’s *t*-test). Concomitantly significantly less cells under CR-50 treatments were counted outside the main tissue mass (Cells Out) compared to CTR (*P < 0.01; Student’s *t*-test). Scale bar = 100 μm in **(C)** (same magnification **A,D**). Scale Bar = 50 μm in **(D)**.

**Figure 2:**
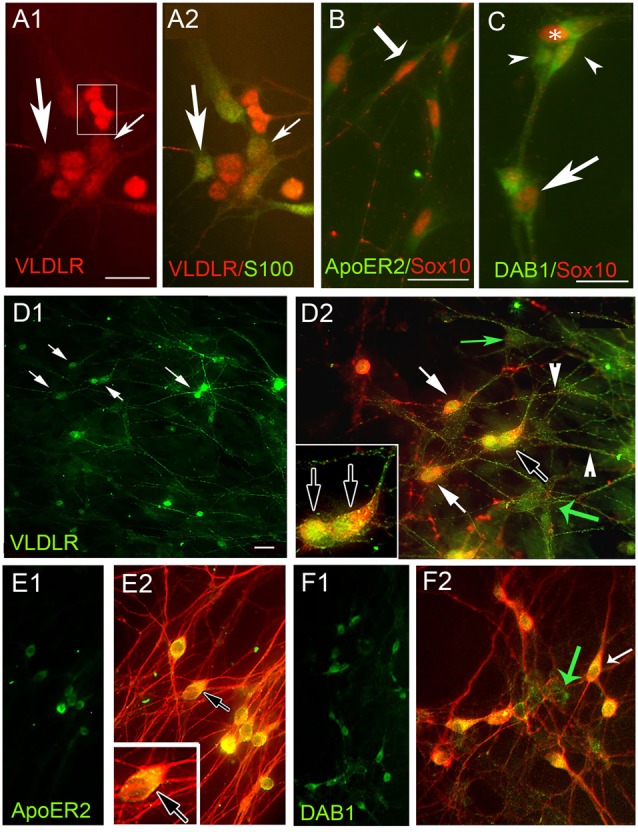
Olfactory ensheathing cells (OECs) and GnRH neurons express Reelin receptors apolipoprotein E receptor 2 (ApoER2), very low-density lipoprotein receptor (VLDLR) and the adaptor protein Disabled 1 (Dab1) in explants. **(A–F)** Periphery of explants double-labeled for OECs **(A–C)** and GnRH cells **(D–F)** with indicated antibodies. **(A)** VLDLR (red) is expressed by OECs (green, S100, arrows). In addition, VLDLR highlighted positive cells associated with OECs (boxed area **A1**) that did not stain for OEC markers. OECs (**B,C** Sox10, red) co-expressed ApoER2 (**B**, green, example shown by arrow) and Dab1 (**C**, green, example shown by arrow). Sox10 negative Dab1 positive cells (arrowheads) were associated with OECs. GnRH cells (**D–F**, red, white arrows), co-expressed VLDLR (**D**, green) ApoER2 (**E**, green) and Dab1 (**F**, green). Numerous non-GnRH positive VLDLR cells (**D2**, green arrows) and Dab1 cells (**F2**, green arrow) were detected, consistent with OEC expression described above. TUJ1 was used to stain both GnRH cells and olfactory axons in **(E,F)**. Although GnRH cells co-expressed ApoER2, olfactory fibers were devoid of staining (no fibers detected in **E1** and fibers only red in **E2**). Dab1 was absent from the majority of olfactory fibers as well **(F)**. Scale Bar = 100 μm in **(A–C)**. Scale Bar = 50 μm in **(D1,E1,F1)**; 100 μm in **(D2,E2,F2)**, double labeled images of **(D1,E1,F1)**, respectively. Insets in **(D2,E2)** correspond to black arrow in lower magnification.

**Figure 3:**
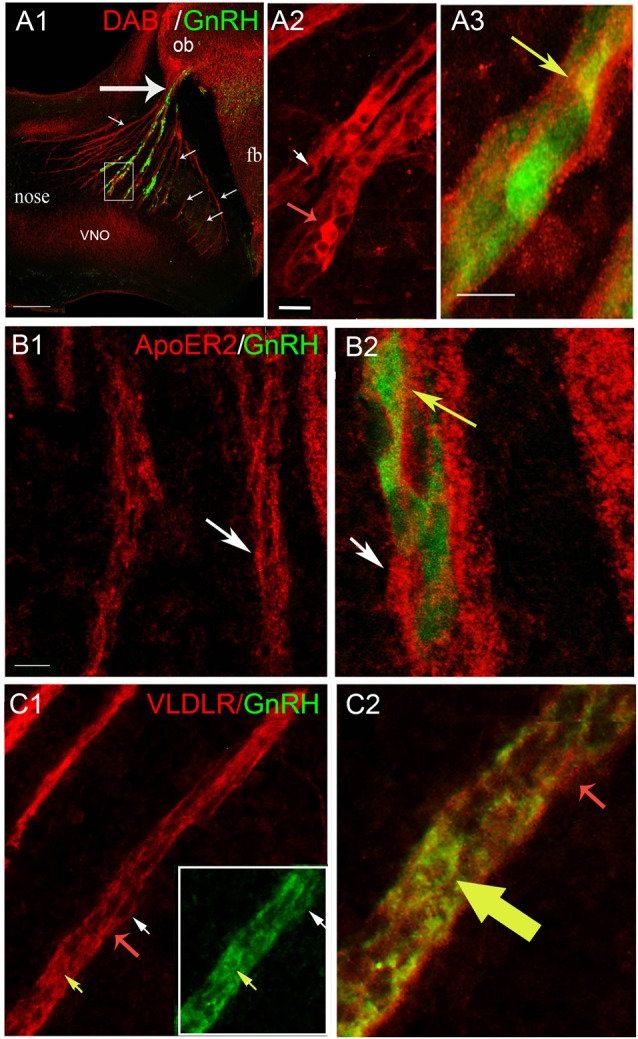
GnRH neurons express Dab1, ApoER2 and VLDLR during of mouse embryonic development. **(A–C)** Sagittal section of E12.5 mice (16 μm) immunostained with indicated antibodies; GnRH-GFP using green fluorescent protein (GFP) antibody. **(A)** Low magnification of embryonic head stained for Dab1 (red) and GnRH-GFP (green) shows tracks in nasal region (small arrows; with GnRH cells from the vomeronasal organ (VNO), and without GnRH cells from the olfactory epithelium), crossing to the nasal/forebrain junction (large arrow); ob, olfactory bulb; fb, forebrain. High magnification of area boxed in **(A)**. Double labeling for Dab1 (red, **A2**) showed expression in some GnRH-GFP cells (green, yellowed merge, **A3**). Dab1-immunoreactivity was often confined to the rim of the GnRH cells but co-expression in leading processes was detected (**A3**, yellow arrow). In addition, Dab1 positive non-GnRH cells were located along the axonal tracts (**A2**, red arrow). **(A3)** is higher magnification of area shown by white arrow in **(A2)**. **(B)** Immunofluorescence for ApoER2 (red) and GnRH-GFP (green) revealed co-expression in GnRH cells crossing the nasal mesenchyme (**B2**, higher magnification of **B1**, merged, yellow arrow, white arrow is same location in both **B1**,**B2**). ApoER2 was also detected on other elements along the migratory pathway (**B**, white arrows). **(C)** Double labeling for VLDLR (red) and GnRH-GFP (green) indicated that the majority of GnRH neurons expressed VLDLR (Boxed area in **C1**, shown at higher magnification, merged in **C2**, yellow and red arrows indicate same location in **C1**,**C2**). As with Dab1 and ApoER2, VLDLR was also detected on other elements along the migratory pathway (**C**, red arrows). Scale bar = 200 μm in **(A1)**, 20 μm in **(A2)** (same in **B1,C1**) and 40 μm in **(A3)** (same in **B2,C2**).

**Figure 4:**
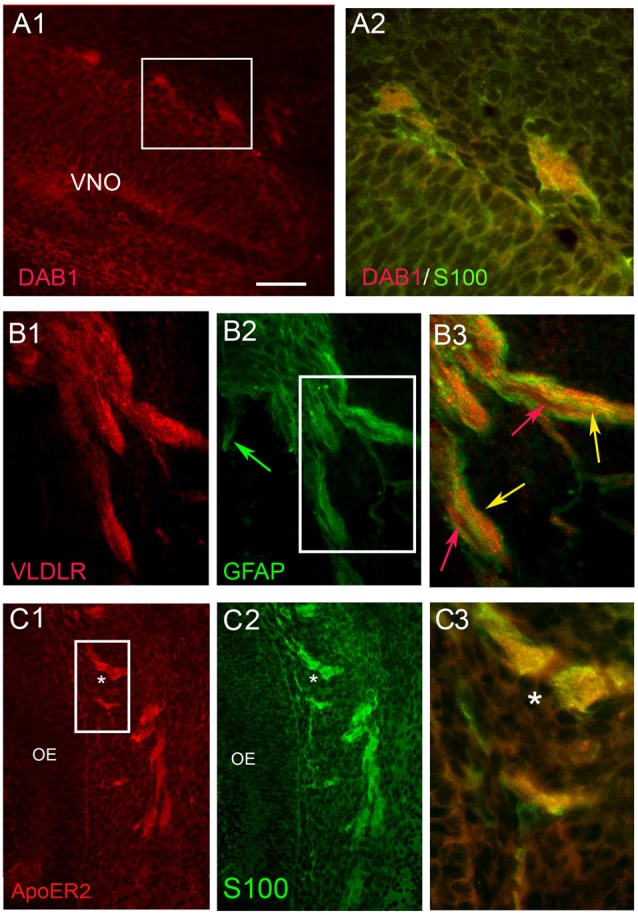
OECs express Dab1, ApoER2 and VLDLR during mouse embryonic development. Double label experiments were performed at stages E12.5-E14.5. OECs, identified by S100 or GFAP (green), expressed Dab1 **(A)**, VLDLR **(B)** and ApoER2 **(C)**. Scale Bar in **(A1)** = 200 μm in **(A1,B1,2,C1,2)**; 100 μm in **(B3)** and 50 μm in **(A2,C3)**. Boxed areas in **(A1,B2)** show locations of **(A2,B3)** respectively. In row **(B)**, red and green arrows show examples of elements expressing single label, while yellow arrows indicate examples of double-labeled elements. Asterisk in **(C1–3)** reference same location. OE, olfactory epithelium.

### Nasal Cartilage, GnRH Cell and OEC Isolations and RNA Extraction

Timed pregnant NIH Swiss mice were obtained according to an NINDS ACUC approved protocol. Pregnant mice were euthanized at embryonic day 11.5 and embryos harvested as for making explants (described above). Briefly, embryonic heads were removed, and the nose isolated from the brain by a single coronal cut. From this tissue, the midline nasal cartilage and surrounding mesenchyme was isolated. The midline tissue was then cut in half along the horizontal plane and “top” or “bottom” transferred to a tube on ice until all embryos from a single litter were processed. Tubes were then placed on dry ice until RNA extraction. One or two litters (average eight animals per litter) were processed for RNA isolation together. GnRH single cell isolation and single-cell RT-PCR were performed as described previously (Kramer et al., 2000). Briefly, nasal explants, were washed twice with PBS (without Mg^2+^ or Ca^2+^) and placed in 2 ml of the same solution. Single GnRH cells were identified under a Carl Zeiss inverted microscope (Zeiss, NY, USA) by their bipolar morphology, position and association with outgrowing axons. At two time points (4 and 10 days *in vitro* div), single GnRH cells were isolated from nasal explants and cDNA libraries made for both single cells and midline nasal cartilage (Kramer et al., 2000).

Primary cultures OECs were generated from olfactory bulbs of up to 1-week-old neonatal mice. Cells are purified by the differential cell adhesion method (Nash et al., 2001) and cultured for up to 2 weeks. Briefly, olfactory bulbs were collected and degraded in an enzyme mix (hyaluronidase (Sigma), dispase I (Sigma), collagenase type 4 (Worthington), DNAse Worthington; Au and Roskams, 2002) for 35 min (37°C) with constant agitation. Cells were strained through a 40 μm cell strainer to remove non-dissociated tissue pieces and then washed with media (Dulbecco’s minimum essential media (Gibco) and Ham’s F-12 (Gibco), at a 1:1 mixture with 10% fetal bovine serum (Gibco), 1% antibiotic mixture PSN, Gibco) to remove enzyme residues. Re-suspended cell solution (4 × 10^6^ viable cells/flask) was seeded into uncoated T75 flasks for 18 h to remove fast attaching fibroblasts. The supernatant of the first flask was seeded into another uncoated flask for up to 36 h to allow attachment of astrocytes. The final supernatant was seeded onto poly-L-lysine (Sigma)-coated flasks to grow primary OECs. Media was changed every 2–3 days. Cells were trypsinized, four coverslips seeded for staining to confirm phenotype, and the rest of the cells were processed for RNA extraction (RNAqueous kit; Ambion). Following extraction, cDNA was created using Moloney murine leukemia virus reverse transcriptase (Superscript™ III; Invitrogen). The purity of OECs was determined to be more than 90% based on p75 and S100B immunostaining.

### Microarray Analysis

For microarray experiments on GnRH cells and midline nasal cartilage, all cDNAs were further processed (labeled and hybridized) by the DIRP NIH microarray core facility. GeneChip Mouse Genome 430 2.0 arrays (Affymetrix: Santa Clara, CA, USA) were used. The microarrays were checked for noise and outliers using custom R scripts, including covariance-based principal component analysis (PCA), correlation heat maps, LOWESS analysis and clustering. The data were normalized such that the average expression of genes in all chips was similar and log transformed. Since levels of receptors were generally an order of magnitude lower than housekeeping genes, receptor expression was analyzed as a group to determine background vs. positive signals. Data is given as maximum Robust Multiarray Averaging (RMA; Izadi et al., 2016). Previous work established that GnRH cells robustly expressed CXCR4 chemokine receptors (Toba et al., 2008; Casoni et al., 2012). The RMA value for CXCR4 receptors (5.7) was used for comparison of expression VLDL, ApoER2 and Dab1 values.

### PCR on Single GnRH Cells and Primary OECs for Dab1

Poly(A) amplified cDNA libraries were created from individual GnRH cells as described above. The phenotype of each single cell cDNA pool was confirmed by PCR for GnRH. The purity of the OECs was confirmed by PCR for p75 (OEC positive: melting temp at 56°C; product size: 264; F-5’ TGCAATTAGTAGAAGGACCCCACC 3’; R-5’ TACACA GGATAGCAAAGGGGA 3’) and myelin basic protein (OEC negative: melting temp at 56°C; product size: 282; F-5’ GAGACCCTCACAGCGATCCAAG 3’; R-5’ GGAGGT GGTGTTCGAGGTGTC 3’). Both single GnRH cells (*n* = 8) and OEC cDNAs were analyzed for Dab1 transcripts. Primers were designed in the 3’-untranslated region of the gene disabled one within 200 base pairs prior to the polyadenylation site (Dab1, sequence ID # NM_177259.4, melting temp at 55°C; product size: 164; F-5’ TGACTGTTGCAGTCCGTTTC, R-5’ TGTAACCGTTTTACATGGCGTG). All primers were screened using BLAST to ensure specificity. For each reaction, 30.5 μl H2O, 5 μl 10× PCR buffer, 4 μl 25 mM MgCl2, 5 μl deoxynucleotide mix (Life Technologies; 25 μl of each 100 mM deoxynucleotide, 900 μl H2O), 2 μl 6.25 μM forward primer, 2 μl 6.25 μM reverse primer, and 0.5 μl AmpliTaq Gold (Life Technologies) were added to 1 μl template cDNA. PCR was performed as following: initial 10-min denaturation (94°C); 40 cycles with denaturation 30 s (94°C); annealing 30 s (see above) and extension 2 min (72°C); followed by a 10-min post elongation at 72°C. Amplified products were run on a 1.5% agarose gel. Specific bands of the predicted size were observed in control total brain, whereas no bands were seen in water.

### Functional Assays in Nasal Explants

To determine the function of Reelin in the developing GnRH/olfactory systems, nasal explants were used. In the first experiment two groups were compared, explants maintained in 1.5 ml of SFM containing 1 μg of either CR-50 monoclonal antibody (100 μg IgG in 100 μl PBS, MBL, International Corp., an antibody known to block the effects of Reelin (Ogawa et al., 1995; Nakajima et al., 1997; Zhao et al., 2004) or mouse IgG (control), added twice daily. Antibodies were diluted in 200 μl SFM, 200 μl removed from explant and antibodies added. Explants were treated at 3, 4, 5 div and fixed in 4% of formaldehyde at 6 div. Immediately after fixation, explants were processed immunocytochemically for GnRH neurons (control *N* = 12, treated *N* = 13), olfactory fibers (staining for peripherin, control *N* = 6, treated *N* = 5) or OECs (staining for S100 or Sox10, control *N* = 5, treated *N* = 3). GnRH cell migration, olfactory axon outgrowth and OEC migration were quantified. For GnRH cells and OECs, stained images were taken on a Nikon Eclipse E800 (Nikon USA, Melville, NY, USA) equipped with an ICCD camera (Retiga, Qimaging, Burnaby, BC, Canada). The digital acquisition was made through IPLab software (IPLab Spectrum, Scanalytics Inc., Rockville, MD, USA). The number of immunopositive cells was counted inside the main tissue mass (cell in, just for GnRH cells) as well as outside the main tissue mass in the periphery of the explant (cell out, both GnRH and OECs; Fueshko et al., 1998) using ImageJ software (Rasband, W.S., ImageJ, U. S. National Institutes of Health, Bethesda, MD, USA^1^, 1997–2008). The main tissue mass contained the nasal pit/olfactory epithelial region, surrounding mesenchyme, and nasal midline cartilage (NMC). The periphery refers to the area surrounding the main tissue mass into which cells had spread and/or migrated. For olfactory axons, the distance, as measured by 200 μm zones in which the bulk of the fibers or cells were located, was recorded. Groups were compared using Mann-Whitney, *p* < 0.05 for significance.

The second experiment, similar to those described above, was performed using three groups: CR-50, G10 (an antibody against Reelin previously used for immunocytochemistry which has not been reported to block function, 100 μg IgG in 100 μl PBS, Millipore) and CR-50 + Reelin (1 μg Cr-50 + 5 μg Reelin/1.2ml; Recombinant mouse reelin protein 100 μg/ml in PBS, R&D Systems). The position of cells in the explant periphery was expressed relative to the tip of the cartilage i.e., the larger the number is, the further the cells migrated from the tissue mass. For each treatment, *N* = 5. The cell numbers were compared with a two-way ANOVA, followed by *post-hoc* Tukey’s multiple comparisons test. The spatial distributions of the cells between treatments were compared using the non-parametric Kolmogorov-Smirnov (KS) test (*p* value < 0.001). To identify what proportion of the cells contributed most to the significance, cells were distributed in eight bins (60–120-μm wide, the outliers were combined in the last bin) and chi-square tests were performed (*p* value < 0.001).

### Analysis of *Reeler* Mice

Serial sagittal sections (16-μm thickness) from *reeler* and wildtype (WT) E13.5/E14.5 mice (B6C3Fe a/a-Reln/J) were cut and stained for GnRH, OECs or olfactory sensory axons. Quantitative analysis of GnRH neurons was performed as a function of location with GnRH cells counted in three regions (nasal compartment, nasal/forebrain junction and brain). GnRH cell number/region and total number were analyzed using an ANOVA; *p* < 0.05 for significance (no difference was found as a function of age (*p* = 0.43) thus E13.5 and E14.5 were grouped; *N* = 5 control and 7 *reeler*). Serial coronal sections (16 μm, 4 series) from ≥PN 26 *reeler* and WT mice (*N* = 4 each, B6C3Fe a/a-Reln/J) were cut and labeled for GnRH as described above (GnRH immunoreactivity visualized using DAB substrate). Total number of GnRH cells were calculated and combined to give group means ± SEM. For examination on OECs and olfactory sensory axons, sections were stained with GFAP, Sox10 or p75 (for OECs), Tyrosine hydroxylase (glomeruli) or peripherin (olfactory axons). The section containing fibers entering the olfactory bulb were photographed, imported in ImageJ, digitized and density of fibers measured. Values were analyzed using an ANOVA; *p* < 0.05 for significance.

## Results

### Reelin Immunodepletion Disrupts Migration of GnRH Neurons and OECs

A previous report suggested that Reelin plays a role in guiding GnRH neurons via a non-conventional signaling pathway (Cariboni et al., 2005). The present study was initiated to characterize this non-conventional pathway. Primary GnRH neurons maintained in nasal explants allow one to examine ligand/receptor/signaling pathways in a controllable environment (Fueshko and Wray, 1994; Klenke and Taylor-Burds, 2012). GnRH cells migrate in this model system, recapitulating many of the events seen *in vivo*. Expression of *reelin* transcript and protein in the nasal midline mesenchyme has been detected at E12.5 in mouse (Schnaufer et al., 2009). Since nasal explants are generated from E11.5 mice, the presence of *reelin* transcript and protein was first verified. Microarray analyses were conducted on material obtained from nasal midline cartilage. For this purpose, tissue samples were harvested at E11.5, a stage corresponding to the beginning of the GnRH migratory process. *Reelin* transcript was identified in this midline screen (5.47–6.22 mean RMA values) and immunocytochemistry confirmed expression of the protein in nasal explants (Figure 1). At 3–4 div, GnRH neurons have migrated from the inner tissue mass out into the periphery (Wray, 2010). At this stage, Reelin immunoreactivity was detected in a group of cells at the tip of the NMC, where GnRH cells emerge (Figures 1A,B). To determine whether perturbation of Reelin signaling would alter GnRH neuronal migration, explants were chronically treated from 3–6 div with CR-50, a Reelin antibody that recognizes the full-length protein and interferes with Reelin homopolymerization thereby blocking the effects of Reelin (Ogawa et al., 1995; D’Arcangelo et al., 1997; Cariboni et al., 2005). The concentration of the antibody used to neutralize the activity of Reelin (1 μg/1.5ml media) was similar to that used by others in *in vitro* experiments (Cariboni et al., 2005). Treatment of 3–6 div was chosen because it is the temporal window during which large numbers of GnRH neurons migrate from the inner tissue mass into the periphery of the explant (Fueshko and Wray, 1994). Treated and control explants were fixed at 7 div and stained for GnRH (Figures 1C,D). No significant differences were found in total number of GnRH cells present in control and CR-50-treated explants, arguing against mitogenic or survival effects of Reelin on GnRH neurons. However, application of Reelin antibody severely stunted the migration of GnRH neurons, with significantly more GnRH cells remaining on the inner tissue mass of the explants as compared with controls (Cells In, Figure 1E; *p* < 0.01). In CR-50 treated explants, clusters of GnRH neurons were detected along the midline cartilage (Figure 1D, arrows). Concomitantly, fewer GnRH neurons were located in the periphery (Cells Out, Figure 1E). To determine whether Reelin receptive cells might be altering GnRH cell migration indirectly, rather than via a non-conventional signaling pathway, two other major components of this system were evaluated, olfactory sensory neurons and OECs. It is known that perturbation of olfactory axon extension can alter GnRH cell migration (Wray, 2010). In addition, a guidance role for OECs has been suggested (Cummings and Brunjes, 1995) and impaired maturation of OECs has been linked to olfactory axon tangle formation and misrouting, and defective GnRH neuron migration to the brain (Forni and Wray, 2012). Thus, olfactory sensory neurons and/or OECs could be responding to decreased Reelin and indirectly decreasing GnRH cell movement during treatment. Treatment of explants was performed as described above and olfactory axon extension examined after immunostaining with peripherin and OEC location examined after immunostaining with BLBP. In these experiments, the explant periphery was divided into 200 μm zones extending from the tip of the cartilage (Giacobini et al., 2004), and the zone with the bulk of the sensory axons or OECs recorded. On average, olfactory axons extended 6.3 zones (1.26 mm) in control explants (*N* = 6) and 5.6 zones (1.12 mm) in treated explants (*N* = 5). Although extension was less in treated explants, no statistical difference was found (*p* = 0.36; Mann-Whitney). In contrast, a significant decrease was detected in migration of OECs (Control: 1.13 mm, *N* = 5; treated: 0.84 mm, *N* = 3; *p* = 0.05; Mann-Whitney). These data indicated that Reelin signaling was altering movement of both GnRH cells and OECs. To evaluate whether OECs expressed the canonical signaling Reelin components and might be altering GnRH cell migration indirectly, immunocytochemistry was performed.

### Reelin Receptors and Dab1 Are Expressed by OECs and GnRH Neurons

Immunocytochemistry for VLDLR, ApoE2 and Dab1 in nasal explants showed co-expression in OECs (Figures 2A1–2,B,C respectively). In addition, these experiments revealed that cells associated with OECs were also positive for these markers (boxed area in 2A1, arrowheads 2C). Based on their location and morphology these cells appeared to be GnRH neurons and subsequent staining (for both GnRH (Figure 2D) and TUJ1 (a neuron-specific marker that strongly labels olfactory and vomeronasal structures, as well as GnRH neurons (De Carlos et al., 1996; Roskams et al., 1998; Figures 2E,F), revealed that the majority of GnRH-positive cells co-expressed VLDLR (Figures 2D1,D2; arrows), ApoE2 (Figures 2E1,E2) and Dab1 (2F1,F2). As expected, non-GnRH cells, positive for all three markers were detected (Figures 2D2,F2, green arrows), consistent with both GnRH cells and OECs expressing the Reelin canonical signaling pathway components. In contrast, olfactory axons exhibited VLDLR staining (green fibers in Figure 2D2; arrowheads) but appeared negative for ApoER2 (Figure 2E) and Dab1 (Figure 2F). To confirm expression of these signaling molecules in GnRH neurons, microarray data previously generated in our lab was used (Kramer et al., 2000; Giacobini et al., 2008) and single cell PCR performed. Microarray values for these receptors was compared to the chemokine receptor CXCR4, known to be robustly expressed by GnRH cells (Toba et al., 2008; Casoni et al., 2012). The RMA value for CXCR4 was 5.7. The RMA values for VLDLR, ApoE2 and Dab1 were 8.9, 5.9 and 5.0, respectively, indicating robust transcript levels for both VLDLR and ApoE2. Since Dab1 had the lowest microarray chip value (log scale), and is essential for the canonical Reelin pathway, presence of its transcript was verified by PCR in both OECs and GnRH cells (Supplementary Figure S1).

**Figure 5:**
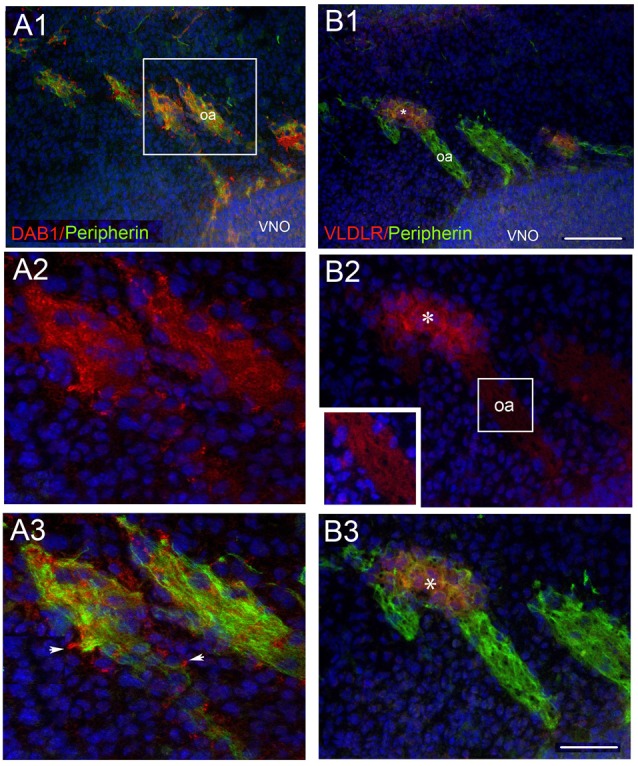
Olfactory sensory neurons express Dab1 and VLDLR during mouse embryonic development. Sagittal sections of E14.5 mice immunostained with indicated antibodies. Olfactory axons identified by antibodies to peripherin (green). Dab1 (**A1–A3**, red), if expressed on olfactory axons, was at a level much lower than that of surrounding OECs and GnRH cells (**A3**, red structures still detected, white arrowheads). VLDLR expression **(B1–B3)** was detected on the axonal tracks (**B2** inset), but also at a level lower than that of surrounding OECs and GnRH cells (Large cluster of VLDLR positive migratory cells is indicated by asterisk). Scale bar in **(B1)** = 25 μm (same in **A1**). Scale bar in **(B3)** = 50 μm (same in **A1,A2,B1**).

Since only 5% of GnRH neurons in E18 rat brains was reported to co-express ApoER2 (Cariboni et al., 2005) and none of the GnRH neurons were reported to be immunoreactive for Dab1, experiments were performed at developmental stages when GnRH cells and OECs are actively migrating (E12.5-E14, Figures 3–5), to ensure that expression of the Reelin canonical signaling pathway molecules in GnRH neurons and OECs maintained in explants reflected that occurring *in vivo*.

Co-expression of Dab1 in some migrating GnRH cells (Figure 3A1) was detected. In addition, many Dab1 positive non-GnRH cells were intermingled with GnRH cells along these tracts (Figure 3A2, red arrow). Immunofluorescence (Figures 3B1,B2) also revealed low levels of co-expression of GnRH and ApoER2 (Figure 3B2, yellow arrow). As with Dab1, other elements along the migratory pathway were also positive for ApoER2 (Figure 3B, white arrows). Double-labeling for GnRH and VLDLR (Figure 3C) indicated that most GnRH cells robustly co-labeled with VLDLR (Figure 3C, yellow arrow). Consistent with earlier data (Schnaufer et al., 2009), VLDLR was not detected in cells in the OE/VNO but was found on non-GnRH cells along the tracks (Figure 3C, red arrows) as well as potentially in the axonal tracks themselves. Examination of Reelin signaling molecules in OECs *in vivo* (labeled with S100 or GFAP) gave similar results to that found in explants. The majority of OECs along the migratory tracts expressed Dab1, ApoER2 and VLDLR (Figure 4) suggesting canonical Reelin signaling can impact OEC migration, as well as GnRH neuronal migration. It was unclear whether cells in the olfactory and vomeronasal organ epithelium expressed low levels of Dab1 or VLDLR (Figures 4A, 5, VNO). Dab1, if expressed on olfactory axons, was at a level much lower than that of surrounding OECs and GnRH cells (Figure 5A, red structures still detected, white arrowheads). VLDLR expression was detected on the axonal tracks (Figure 5B2), but also at a level lower than that of surrounding OECs and GnRH cells (Figure 5B, asterisk). ApoER2 was not detected on cells in the sensory epithelium or on outgrowing axons. Thus, the *in vitro* and *in vivo* expression patterns demonstrate that OECs and GnRH neurons express the Reelin signaling pathway as they migrate into CNS, supporting a role for Reelin as a guidance cue for early migratory GnRH cells and/or for developing OECs. At E18, Cariboni et al. (2005) in fact, reported a decrease in the GnRH cell number in the brain of *reeler* mice compared to WT. To investigate the functional relevance of Reelin in the early development of the GnRH system, OEC development and GnRH cell migration was evaluated in *reeler mice*.

### OEC Development and GnRH Neuronal Migration Is Normal in *Reeler* Mutants

**Figure 6:**
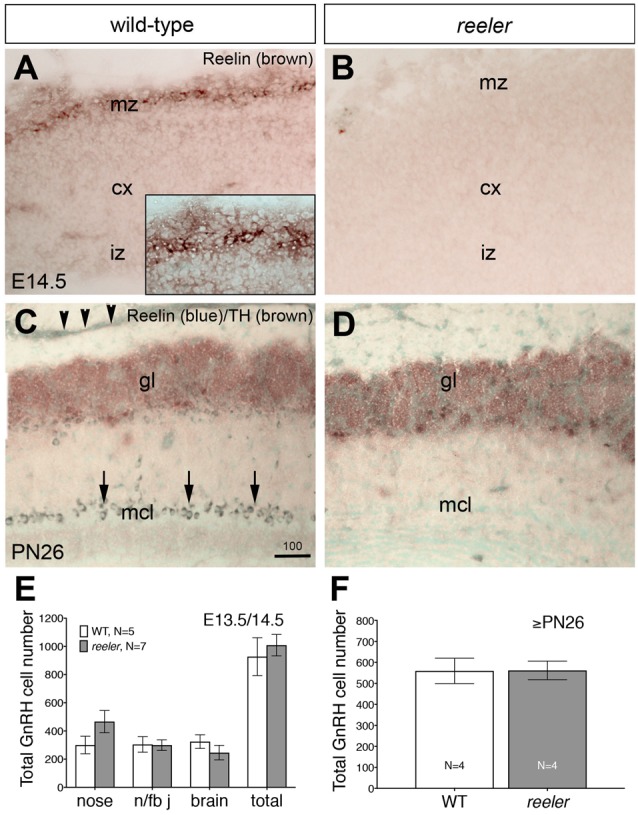
GnRH neuronal migration is normal in *reeler* mice. **(A–D)** Sagittal sections of E13.5/14.5 and ≥PN26 wildtype (WT) and *reeler* mice (B6C3Fe *a/a-Reln/J)* immunostained for indicated antibodies. **(A,B)** Cajal-Retzius cells are among the earliest neurons to be generated in the mammalian neocortex (cx) where they occupy positions near the pial surface, in the marginal zone (mz). In WT E14.5 mice these cells are Reelin-immunoreactive (**A**, high power view shown in inset) whereas the cerebral cortex of *reeler* mice did not react with this antibody **(B)**, although Cajal-Retzius neurons are present (Derer, 1985; Ogawa et al., 1995). **(C)** Sagittal OB section of PN26 WT mouse immunostained for Reelin (blue) and tyrosine hydroxylase (TH; brown). Strong Reelin-immunoreactivity is detectable in the mitral cell layer (mcl; arrows) as well as in the olfactory nerve layer (arrowheads). TH-immunostaining identifies the glomerular layer (gl) of the OB. **(D)** In the OBs of *reeler* mice, Reelin is totally absent. **(E)** Analysis of GnRH neurons location at E13.5/E14.5 revealed no differences in the total number of cells or in the distribution of the GnRH-positive cells across their migratory route in WT and in *reeler* embryos. The areas of analysis for GnRH neuron location along the migratory pathway were the nasal compartment (nose), the nasal/forebrain junction (n/fb j) and the brain, respectively. **(F)** Quantitative analysis of GnRH neuronal population in ≥PN26 brains revealed no differences in the Reelin mutants as compared to WT (*P* > 0.1). Quantitative data are mean cell number ± SEM. Bar in **(C)** = 100 μm (same in **D**).

Previously described phenotypes of *reeler* mice were verified in our specimens before subsequent analysis (Figures 6A–D). The first *reeler* mice analyzed in this study are characterized by a complete gene deletion of *Reelin* (strain B6C3Fe *a/a-Reln/J*; Jackson Laboratories). Previous studies showed that at E12.5 and E14.5, prominent expression of Reelin by Cajal-Retzius cells was detected just beneath the pial surface of the cerebral cortex corresponding to the marginal zone or the future layer I (Stanfield and Cowan, 1979; Ikeda and Terashima, 1997). The specificity of the *reeler* phenotype was confirmed immunohistochemically. Staining was detected in WT littermates but no Reelin-immunoreactive cells were detected in the marginal zone of the embryonic cortex (Figures 6A,B; Katsuyama and Terashima, 2009) or mitral cell layer of the OB of ≥PN26 mutant mice (Figures 6C,D; see arrows), confirming the mutant genotype.

**Figure 7:**
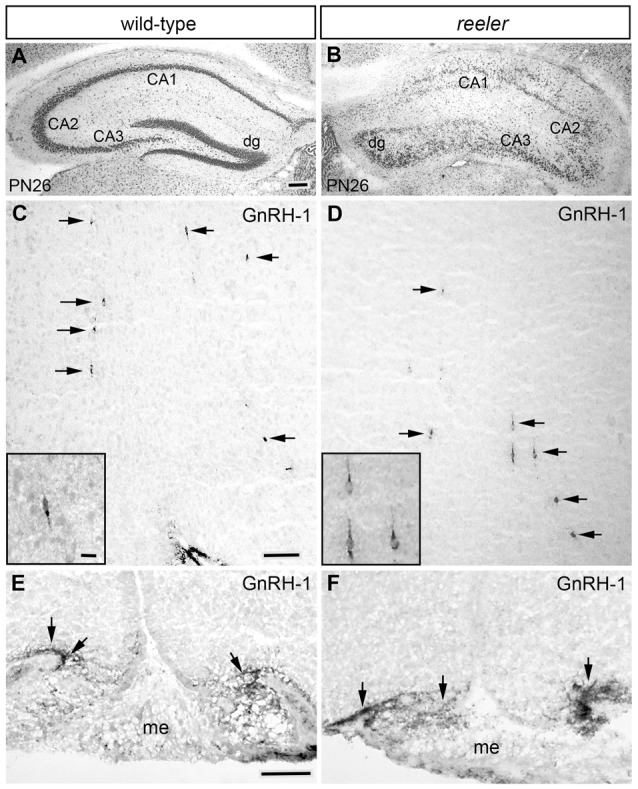
Distribution of GnRH neurons is not affected in the brain of adult *reeler* mice. **(A,B)** Cresyl violet countered coronal sections through the hippocampus showing the cytoarchitecture of the normal **(A)** and *reeler* (B6C3Fe *a/a-Reln/J*; **B**) mice. As expected, the distinct laminar structure of the hippocampus proper (i.e., CA1, CA2 and CA3) and dentate gyrus (dg) of the normal mouse **(A)** was disrupted in the *reeler* mouse **(B)**. **(C–F)** Photomicrographs showing GnRH immunoreactivity (black arrows) in the hypothalamus of the same PN26 WT **(C)** and *reeler* mice **(D)** analyzed in **(A,B)**. No differences in the morphology of the GnRH cells or their terminal fields, the number of cells or their distribution was observed between WT and *reeler* mice. **(E,F)** Photomicrographs showing similar GnRH-immunoreactivity in coronal sections of WT **(E)** and Reelin null mice **(F)** median eminence (me). No observable alterations in GnRH staining was present. Bar in **(A)** = 100 μm (same in **B**). Bar in **(C)** = 100 μm (same in **D**). Bar in **(C)** inset = 25 μm (same as inset in **D**). Bar in **(E)** = 50 μm (same in **F**).

Examination of embryos at E14.5 and adult brains from these *reeler* mice showed no obvious differences in ingrowth of olfactory axons (peripherin staining, data not shown), presence of OECs (Sox10, GFAP and S100 staining, data not shown) or glomeruli formation (tyrosine hydroxylase staining, data not shown) as compared to WT. In addition, Reelin-null mice at E13.5/14.5 revealed a normal complement of GnRH neurons in all anatomical regions analyzed and the total number of GnRH cells was similar between mutant mice and controls (Figure 6E). Since these data were in contrast to that reported by Cariboni et al. (2005), a later time point, ≥PN26 mice, was examined. The total number of GnRH cells in the brain was similar between *reeler* and WT mice (Figure 6F; *p* > 0.05). The distribution of GnRH neurons, the morphology of the cells, as well as their terminals in the median eminence was unaffected when compared to WT mice from the same litter (Figures 7C–F), whereas abnormalities in the DGC layer lamination in the Reelin-null mice were detected as previously described (Figures 7A,B; Stanfield and Cowan, 1979; Förster et al., 2002; Weiss et al., 2003). In the Cariboni study (Cariboni et al., 2005) a different strain of *reeler* mutant mice was used. To insure our data was not mutant line specific, adult animals from the line used in the Cariboni study were obtained. This strain of *reeler* mice (*reeler*/Orleans, Relnrl-Orl) possesses a truncated Reelin protein, which is produced but not secreted (de Bergeyck et al., 1997). In adult Relnrl-Orl mice, the total GnRH cell number was 586 in heterozygous mice (*N* = 2) and 603 ± 99 in mutants (*N* = 3). The two different mutant mouse lines had a similar total number of GnRH neurons (*p* = 0.69). These experiments indicate that the lack of Reelin *in vivo* does not alter development or final position of GnRH neurons when analyzed as early as E13.5.

Since depleting Reelin *in vivo* vs. in nasal explants gave different results, a second set of experiments was performed in nasal explants to validate that the CR-50 antibody was blocking the Reelin pathway. Three explants groups were run using G10 (an antibody with no known functional effects, control), CR-50 (mutant-like) and CR-50 + Reelin (rescue). None of the treatments had an effect on the total number of cells, GnRH neurons or OECs, in the periphery (GnRH: 229 ± 50 [mutant-like]; 247 ± 89 [Control]; 253 ± 85 [Rescue]; OECs: 1351 ± 565 [mutant-like]; 1203 ± 385 [Control]; 1442 ± 315 [Rescue], *N* = 5 in every group). Notably, the distribution of GnRH cells (Supplementary Figure S2) and OECs in the periphery of the explants was similar in the Control and Rescue groups (KS, *p* > 0.001) compared to the Mutant-like group (KS, both *p* < 0.001). Chi-square analysis indicated a greater number of OECs and GnRH cells in the vicinity of the cartilage tip (endogenous Reelin source, 2 first bins) in Mutant-like (OECs: mutant-like: ~22%, Control ~17%, Rescue ~19%; GnRH neurons: Mutant-like ~16%, Control ~9%, Rescue ~9%). These data indicate that exogenous Reelin blocked the effect of CR-50, though GnRH cells were rescued to a greater extent than OECs.

## Discussion

Non-canonical signaling pathways of Reelin remain unclear (Senturk et al., 2011; Bock and May, 2016; Pohlkamp et al., 2016; Talebian et al., 2018). Based on a previous report (Cariboni et al., 2005), GnRH cells were a candidate for studying this phenomenon. However, this report documents the presence of the Reelin canonical signaling pathway (VLDLR, ApoER2 and Dab1) in GnRH cells as they migrate from nasal areas to the brain during embryonic development in mouse. In addition, we document VLDLR, ApoER2 and Dab1 in developing OECs. Chronic perturbation of Reelin in explants generated from E11.5 embryos decreased OEC migration and GnRH neuronal migration but not olfactory axon outgrowth, consistent with the expression data. However, normal migration of GnRH cells was found in two different lines of *reeler* mutant mice and no changes in OECs within the olfactory bulb were detected, suggesting that these two systems are rescued in *reeler* mice at a developmental time point after E11.5.

With respect to the GnRH cells, our data are in contrast to earlier work that found no Dab1 and little Reelin receptor expression in GnRH neurons (Cariboni et al., 2005). However, it should be noted that Cariboni et al. (2005) looked at the expression of these molecules in an immortalized mouse GnRH cell line and *in vivo* in GnRH cells in rats at a later developmental stages (E18). The expression of the full canonical Reelin pathway in OECs has not been previously reported. In agreement with an earlier study (Schnaufer et al., 2009), VLDLR expression was detected in olfactory axons but Dab1 highlighted non-axonal structures (GnRH cells and OECs), suggesting that olfactory sensory neurons do not have a functional canonical signaling pathway for Reelin. In nasal explants, peripherin, which marks olfactory sensory axons, was used to measure olfactory axon outgrowth. Chronic treatment with CR-50 did not significantly alter olfactory axon extension. These data are consistent with a report by Teillon et al. (2003) that found that the vomeronasal nerves were indistinguishable in normal and *reeler* mutant mice. In addition, Wyss et al. (1980) reported no significant alteration in either the layer of olfactory nerve fibers or the glomeruli themselves in *reeler* mutant mice (Wyss et al., 1980). In contrast to olfactory sensory cells, this report identifies that OECs, like GnRH neurons, express VLDLR, ApoER2 and Dab1. Although Antal et al. (2015) examined olfactory placode development in human fetal tissue and performed double immunostaining for Dab1 and S100 protein (which marks OECs), they were not able to conclude anything definitive about Dab1 expression in OECs. Notably in nasal explants, the migration of OECs was examined after chronic treatment with CR-50, and a significant decrease in OEC migration into the periphery was found. These data support our expression data and indicate that a functional Reelin signaling pathway is present in OECs. However, no changes in OECs within the olfactory bulb were observed in *reeler* mice, suggesting again that a rescue event occurs in *reeler* mice at a developmental time point after E11.5.

Examination of Reelin null mice did not show any deficits in the migration of GnRH neurons. Moreover, adult brains from *reeler* mice contained the normal complement and a similar distribution of GnRH cells. These results are in contrast with previous findings revealing a reduction of GnRH-immunoreactive neurons in the hypothalamus of *reeler*/Orleans mice compared to WT controls (Cariboni et al., 2005). Certainly, a plethora of Reelin-null models exists and have been successfully used to investigate the role of Reelin during development, and in the adult in regions of neuronal plasticity (Rice and Curran, 2001; Tissir and Goffinet, 2003; Katsuyama and Terashima, 2009). It is possible that different genetic backgrounds of the *reeler* mutants could result in phenotypes with various degrees of severity depending on the cellular compartment. Thus, the phenotype of the *reeler*/Orleans was analyzed. These two lines differ in that the first is characterized by a complete gene deletion of *Reelin* (B6C3Fe *a/a-Reln/J*; Jackson Laboratories) while the other one (*reeler*/Orleans) possesses a truncated Reelin protein which is produced but not secreted (de Bergeyck et al., 1997). In both lines, similar results were obtained. Normal development of the GnRH system was found, while other brain regions known to be affected in *reeler* mice (i.e., the hippocampus, the developing cortex and the olfactory bulb) were significantly compromised in both transgenic lines. The reasons for the discrepancies between the present study and Cariboni et al. (2005) are unknown. However, we often find variability in immunostaining of GnRH cells shortly after birth and either do counts later in development or verify with *in situs*. In mice, inactivating mutations of Dab1 (*scrambler* mice), and double mutations of ApoER2 and VLDLR generate *reeler*-like phenotypes (Tissir and Goffinet, 2003) but maintained the normal complement of GnRH neurons in the hypothalamic region. These data are consistent with our findings that the GnRH system is normal in *reeler* mutants.

It is clear that the adhesion, pathfinding and the rate of migration of GnRH neurons across the nasal mesenchyme is regulated by multiple factors expressed along their migratory route, including ECM proteins, adhesion molecules, chemokines, guidance molecules and neurotransmitters (Tobet and Schwarting, 2006). Therefore, compensatory mechanisms are likely to exist in the development of the GnRH as well as the olfactory system of Reelin-null mice since no deficits were found in these mice by E13.5. Functional analysis was performed in nasal explants, which are generated at E11.5 and lack brain tissue. These explants have been successfully used for functional studies (Fueshko et al., 1998; Kramer et al., 2000; Giacobini et al., 2004, 2007) since they maintain large numbers of GnRH neurons and OECs, migrating in a similar manner to that observed *in vivo*, as well as directed olfactory axon outgrowth (Fueshko and Wray, 1994). Reelin was expressed in the nasal cartilage of the explants, and the expression paralleled the *in vivo* distribution. When the activity of Reelin was abolished with chronic treatment with the CR-50 antibody, the GnRH cell migratory behavior was significantly altered as well as that of the OECs. Both cell groups did not migrate as far. However, no changes were detected in outgrowth of olfactory axons. The same concentration of this antibody has been previously used to interfere with the aggregation of Reelin and to block its function *in vitro* and *in vivo* (Ogawa et al., 1995; Miyata et al., 1997; Nakajima et al., 1997; D’Arcangelo et al., 1999; Kubo et al., 2002; Cariboni et al., 2005). In addition, we showed that the effects of CR-50 on GnRH cells and OECs could be blocked by addition of exogenous Reelin. These findings support the idea that endogenous Reelin modulates the migratory behavior of the GnRH system either directly and/or indirectly via OECs. We hypothesize that the lack of a gradient, over time, ceased movement of GnRH cells and OECs in explants, and that unknown endogenous cues not present in our explant model but present *in vivo* prior to E13.5 compensate for the disruption in Reelin signaling *in vivo*.

In sum, the data in this report indicate that the Reelin pathway can modulate, but is not essential, for the development of GnRH neurons and OECs, and that both of these cell types possess the necessary components for Reelin signaling through the canonical pathway and as such, are not appropriate models for identifying a non-conventional Reelin signaling pathway.

## Author Contributions

SW conceptualized and designed experiments. LD, EF, PG, AS, HS, SC and FC performed experiments and analyzed data. BH provided reagents and designed experiments. EF, PG and SW wrote the manuscript.

## Conflict of Interest Statement

The authors declare that the research was conducted in the absence of any commercial or financial relationships that could be construed as a potential conflict of interest.

## Footnotes

^1^http://rsb.info.nih.gov/ij/

## Supplementary Material

FIGURE S1 Disabled 1 (Dab1) transcripts are preset in gonadotropin releasing hormone-1 (GnRH) cells and olfactory ensheathing cells (OECs). Top panel: band of the correct size (164 bps) for Dab1 was detected in cDNA produced from eight individual GnRH cells. Bottom panel: The purity of the OECs was confirmed by PCR for p75 (OEC positive, product size: 264 bps) and myelin basic protein (OEC negative, 282 bps). Like GnRH cells, OECs were positive for Dab1 transcript. Br = Brain (positive control), H_2_0 = water (negative control).

FIGURE S2 Migration of GnRH neurons exposed to Reelin blocking antibody is rescued by co-incubation of Reelin. Three explants groups were run to validate that the CR-50 antibody was blocking the Reelin pathway. One group received G10 (an antibody with no known functional effects, control), one group received CR-50 (mutant-like) and the third group received CR-50 + Reelin (rescue). None of the treatments had an effect on the total number of GnRH cell in the periphery (GnRH: 229 ± 50 [mutant-like], 247 ± 89 [Control], 253 ± 85 [Rescue] *N* = 5 for each group). **(A)** The cumulative frequency of the distribution of GnRH cells in the periphery of explants treated with CR-50 and CR-50/Reelin is plotted. Exogenous Reelin blocked the effect of CR-50 (Kolmogorov-Smirnov (KS), *p* < 0.001), allowing GnRH cells to migrate further into the periphery (especially close to the endogenous reelin source which is at the tip of the nasal cartilage 0 on *x*-axis) **(B)**. The cumulative frequency of the distribution of GnRH cells in the periphery of explants treated with G10 and CR-50/Reelin is plotted. No difference between the Control and Rescue groups was found (KS, *p* > 0.001).
