# Supplementary figures and images for: Reelin Can Modulate Migration of Olfactory Ensheathing Cells and Gonadotropin Releasing Hormone Neurons via the Canonical Pathway
Source: Front Cell Neurosci. 2018 Aug 3;12:228. doi: 10.3389/fncel.2018.00228 (PMC6088185; doi:10.3389/fncel.2018.00228)

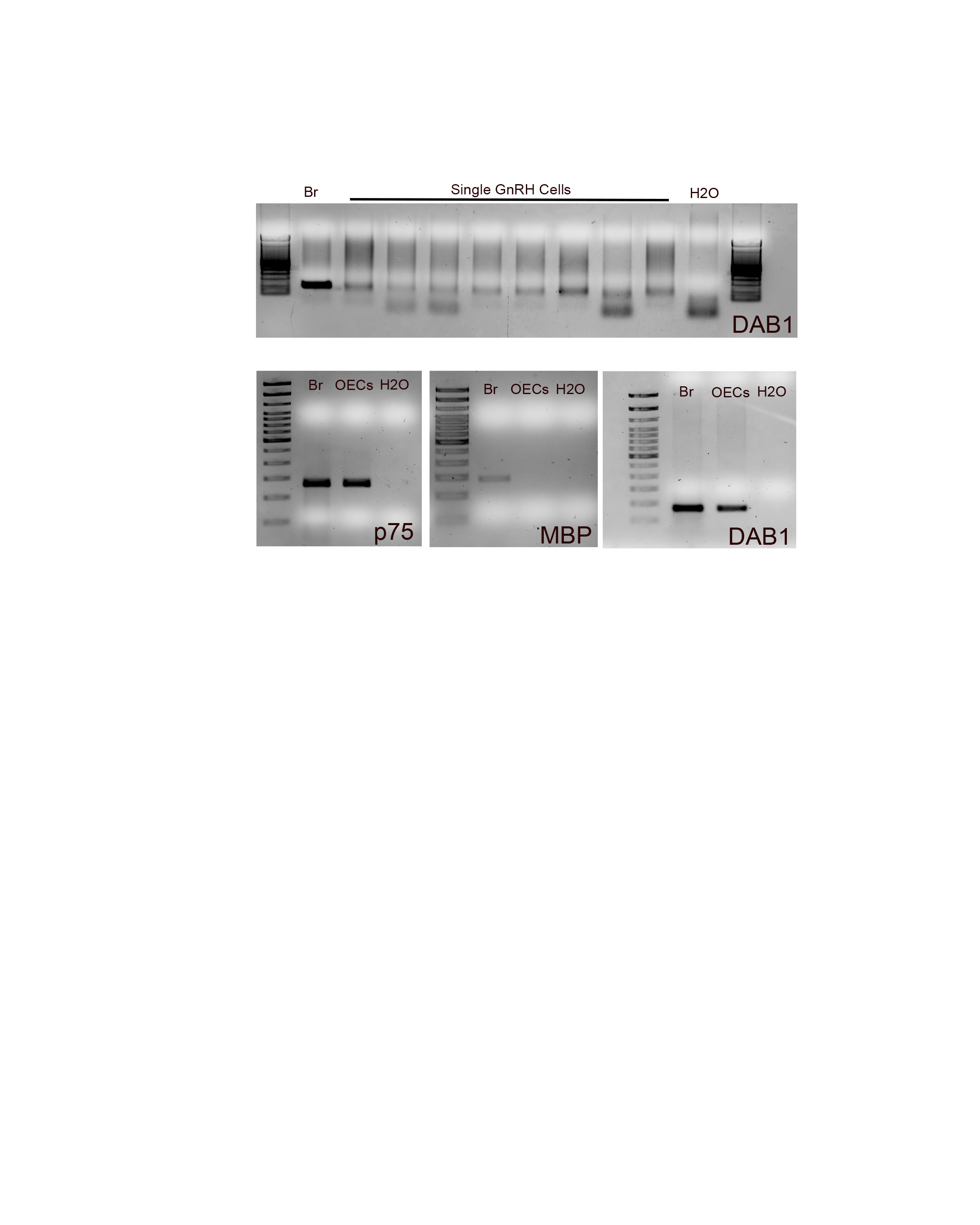

Supplement: FIGURE S1 — Disabled 1 (Dab1) transcripts are preset in gonadotropin releasing hormone-1 (GnRH) cells and olfactory ensheathing cells (OECs). Top panel: band of the correct size (164 bps) for Dab1 was detected in cDNA produced from eight individual GnRH cells. Bottom panel: The purity of the OECs was confirmed by PCR for p75 (OEC positive, product size: 264 bps) and myelin basic protein (OEC negative, 282 bps). Like GnRH cells, OECs were positive for Dab1 transcript. Br = Brain (positive control), H20 = water (negative control). [file Image_1.JPEG]

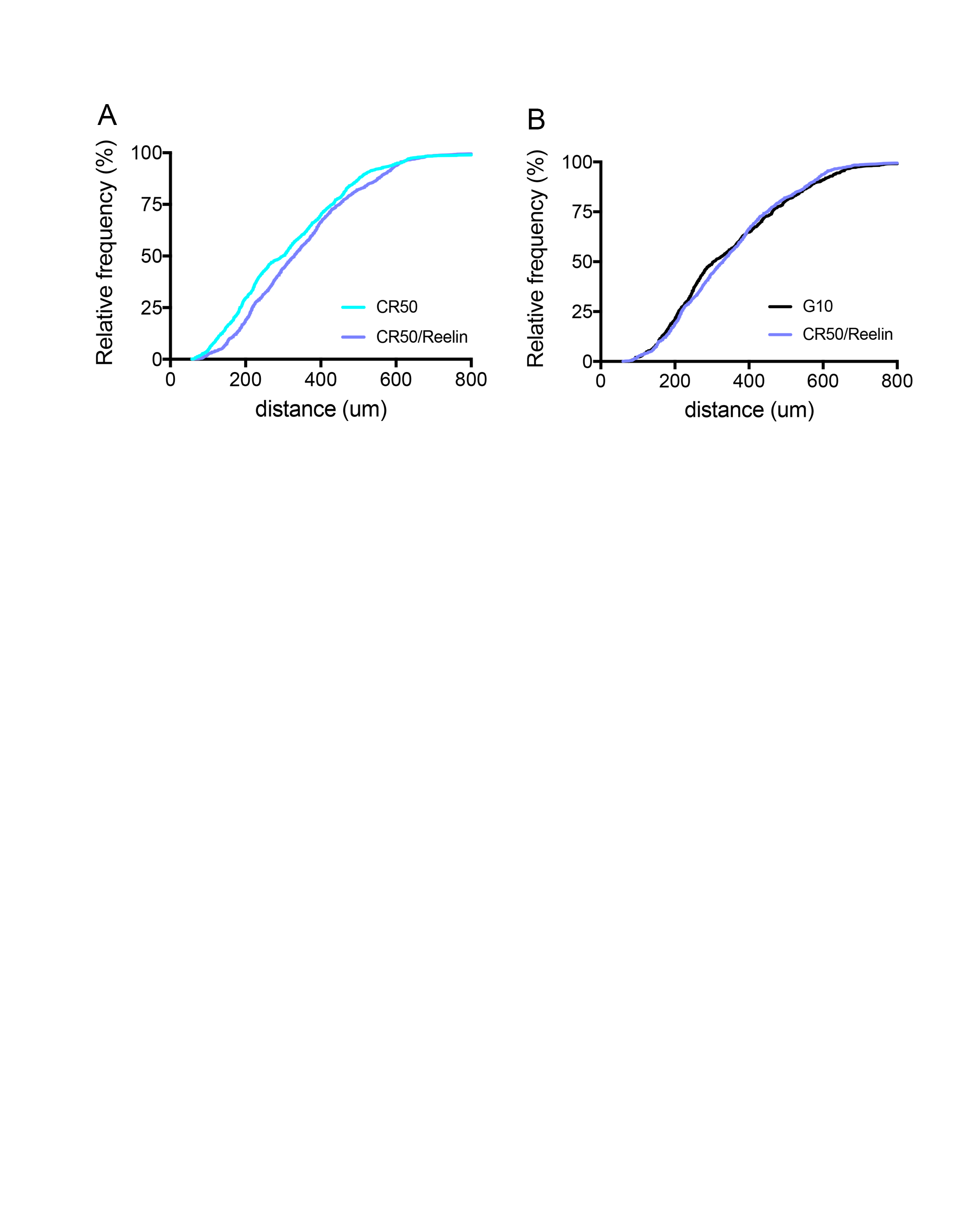

Supplement: FIGURE S2 — Migration of GnRH neurons exposed to Reelin blocking antibody is rescued by co-incubation of Reelin. Three explants groups were run to validate that the CR-50 antibody was blocking the Reelin pathway. One group received G10 (an antibody with no known functional effects, control), one group received CR-50 (mutant-like) and the third group received CR-50 + Reelin (rescue). None of the treatments had an effect on the total number of GnRH cell in the periphery (GnRH: 229 ± 50 [mutant-like], 247 ± 89 [Control], 253 ± 85 [Rescue] N = 5 for each group). (A) The cumulative frequency of the distribution of GnRH cells in the periphery of explants treated with CR-50 and CR-50/Reelin is plotted. Exogenous Reelin blocked the effect of CR-50 (Kolmogorov-Smirnov (KS), p < 0.001), allowing GnRH cells to migrate further into the periphery (especially close to the endogenous reelin source which is at the tip of the nasal cartilage 0 on x-axis) (B). The cumulative frequency of the distribution of GnRH cells in the periphery of explants treated with G10 and CR-50/Reelin is plotted. No difference between the Control and Rescue groups was found (KS, p > 0.001). [file Image_2.TIF]
